# Introgression Among Cultivated and Wild Grapevine in Tuscany

**DOI:** 10.3389/fpls.2020.00202

**Published:** 2020-02-28

**Authors:** Claudio D’Onofrio

**Affiliations:** ^1^Department of Agriculture, Food and Environment, University of Pisa, Pisa, Italy; ^2^Nutraceuticals and Food for Health – Nutrafood, University of Pisa, Pisa, Italy

**Keywords:** chlorotype, domestication, GrapeReSeq 18K Vitis genotyping chip, lambrusque, microsatellite, spontaneous crosses, *Vitis vinifera* subsp. *sylvestris*, *Vitaceae*

## Abstract

Wild grapevine, *Vitis vinifera* L. subsp. *sylvestris* (Gmelin, Hegi) is spontaneous to Europe and common in Tuscany. In this study, wild grapevines were identified in 22 populations from eight locations in Tuscan Maremma (Grosseto and Siena province). The plants were propagated by cuttings, collected in a vineyard, genotyped by nuclear simple sequence repeats (SSRs), chloroplast SSRs and single nucleotide polymorphisms (SNPs), and compared to locally cultivated varieties (*Vitis vinifera* L. subsp. *sativa*) and to non-*vinifera* and non-*vitis* genotypes. The identity analysis revealed that some individuals were redundant genotypes, suggesting natural vegetative propagation. In addition, four of the supposed *V.v. sylvestris* were in fact naturalized *V.v. sativa*. The majority of putative *sylvestris* genotypes had chlorotype A, while the remainder had chlorotype D, as the majority of *Vitis vinifera* subsp. *sativa* cultivated in Italy. Some of the recovered *sylvestris* genotypes appeared to be natural crosses with cultivated grapevine varieties in Tuscany, and their chlorotype suggests a higher pollen flow from *sativa* to the *sylvestris* genotypes than in the opposite direction. In addition, other genotypes appeared to be crosses within *sylvestris*, *sylvestris*-*sativa* or *sylvestris*-*sylvestris* siblings, or equivalent relationships. These relationships suggest a noticeably level of sexual reproductive activities among *sylvestris* and *sylvestris*-*sativa* genotypes. A cluster and structure analysis clearly differentiated the true *sylvestris* from the *sativa*, and the non-*vinifera* or non-*vitis* genotypes, and also highlighted a possible introgression of *sylvestris* into some Italian and French cultivated varieties. The results therefore suggest that, in addition to the primary ancient center of domestication from the Near East to Central Asia, the introgression among cultivated and wild grapevine occurred in other centers of diversification along the migration routes, contributing to the domestication processes, and suggesting that these processes are still ongoing despite the reduction in populations of *sylvestris*. The results also highlight that the GrapeReSeq 18K Vitis genotyping chip are suitable for non-*vitis* genotyping and that the range of SNPs heterozygosity in *sylvestris* appears to be up to 6% less and does not overlap the heterozygosity range of *sativa* genotypes.

## Introduction

The domesticated grapevine (*Vitis vinifera* L. subsp. *sativa*) is the most cultivated fruit crop within the genus *Vitis*. Grapevine domestication occurred about 8000 years ago during the Neolithic Age, in the Near East and the area of northern Mesopotamia and central Asian countries, from the wild grapevine *Vitis vinifera* L. subsp. *sylvestris* (Levadoux, 1956; McGovern, 2003; This et al., 2006; Forni, 2012; Bacilieri et al., 2013). The domesticated grapevines were disseminated from the primary domestication center toward Mesopotamia, the Balkans and the east Mediterranean Basin (at the end of the fifth millenium BC), and toward Sicily and western Europe. They were introduced to central Europe during the first millenium BC (Forni, 2012).

Negrul (1946) divided the domesticated grapevine cultivars into three *proles* (*pontica*, *orientalis*, and *occidentalis*) depending on the geographical distribution and morphological and ecological differences. The *proles orientalis* include grapevine varieties from eastern Georgia, Armenia, and Azerbaijan, the former Soviet republics in Central Asia to the Near East. This *proles* has two sub-proles: *caspica* (the ancient wine grape and Muscat varieties) and the *antasiatica* (table and raisin grape cultivars of a more recent origin). The *proles pontica* comprise the varieties from Georgia to the Balkans, divided into two sub-proles: *georgica* and *balkanica*, respectively. The proles *occidentalis* include the varieties from Western Europe.

Despite the grapevine diffusion from east to west after its first domestication, recent studies on DNA molecular markers provide some evidence of genetic characteristics and introgression from local *sylvestris* individuals in cultivated accessions (Myles et al., 2011). A significant gene flow between *sativa* and *sylvestris* has been observed in regions where these two taxa came into contact (Di Vecchi-Staraz et al., 2008), and additional domestication events that gave rise to the cultivated grapevine have been suggested in Sardinia (Grassi et al., 2006) and also in Spain and France (Riaz et al., 2018). However, the degree to which local *sylvestris* contributed to the domestication of Western European *sativa* cultivars has not yet been well defined.

The wild grapevine is spontaneous from Central Asia to the Mediterranean Basin (Zohary and Hopf, 2000) and is typically found in riparian ravines where it has access to water and can climb into the tree canopies. There is a considerable variety of forms among wild grapevine germplasm, and it can present biotypes of botanical or farming interest. In addition, the wild grapevine is an important source of genetic variation as a source of resilience in breeding programs for the improvement of cultivated vines as well as for dealing with climate change and the increasing demand for sustainable viticulture (Coleman et al., 2009; Ellstrand et al., 2010; Popescu et al., 2013; Meléndez et al., 2016; Marrano et al., 2018).

However, due to the ongoing restriction of its natural habitat, this sub-species is now at risk of extinction. The wild grapevine is undergoing a dramatic regression together with a significant increase in naturalized cultivated accessions, thus endangering the endurance of the wild grapevine in natural ecosystems (Meléndez et al., 2016; Riaz et al., 2018).

Previous investigations have reported that wild vines are widespread in Italy and particularly in Tuscan Maremma (Biagini et al., 2014). This paper presents the results of a project on the safeguarding, genotyping and phylogenetic study of *Vitis vinifera* subsp. *sylvestris* vines in the Tuscan Maremma.

## Materials and Methods

### Plant Material

#### *Vitis vinifera* subsp. *sylvestris*

**FIGURE 1:**
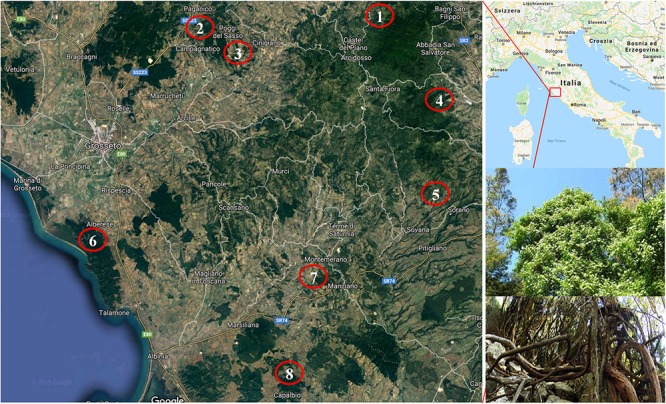
Location of the identification of putative *Vitis vinifera* subsp. *sylvestris*. **(1)** Castiglione D’Orcia; **(2)** Paganico; **(3)** Cinigiano; **(4)** Castell’Azzara; **(5)** Sorano; **(6)** Alberese; **(7)** Manciano; **(8)** Capalbio. In the two inserts in the right bottom: details of the top and bottom sections of a putative *Vitis vinifera* subsp. *sylvestris* vine.

About 150 vines of putative *Vitis vinifera* subsp. *sylvestris* (Gmelin) Hegi were identified in 22 populations (the distances between populations was over 5 km) from eight locations in the provinces of Grosseto and Siena (Figure 1). Most of the vines were identified in typical wild grapevine environments, such as land and woods with a high degree of humidity and the presence of elm, poplar and oak, in coastline zones and valley floors mainly with alluvial and colluvial lands. However, some were collected from locations at the edges of areas that had once been cultivated.

The identification of the wild individual was based mainly on the morphology of leaves: mature leaves with open petiole sinus and upper lateral sinus, absence of prostrate hairs and a low density of erect hairs on the lower side of the blade, and teeth with both sides straight. However, vines with dubious *sylvestris* leaf morphology were also sampled to assess the presence of naturalized *sativa* genotypes and *sylvestris*-*sativa* crosses. When possible, flower sex was also verified *in situ* by selecting diecious plants.

Of the plants identified, 82 were sampled for DNA polymorphism analysis, ranging from 1 to 21 per population (Table 1) and vegetatively propagated by cuttings. The self-rooted plants (usually five for each accession) were planted in a collection vineyard located on the estate of Castello di Collemassari in the Montecucco Sangiovese DOCG (Grosseto Province, Tuscany south coastal area), where flower sex was identified as suggested in a previous study (Stout, 1921; Caporali et al., 2003) and berry color was identified in accordance with OIV descriptor 225 (OIV, 2009).

**TABLE 1:** Location, number of populations per location, number of samples per population, genotypes, sex and berry color of collected putative *Vitis vinifera* subsp. *sylvestris*.

**Sampling**			**Genotypes**		**Sex**				**Berry color**	
			
**Location**	**n. populations**	**n. samples**	**Redundant**	**Unique**	**Female**	**Hermaph.**	**Male**	**Unknown**	**Black**	**White**
(1) Castiglione D’Orcia	1	2	0	2	0	2	0	0	1	1
(2) Paganico	1	2	0	2	1	0	0	1	0	1
(3) Cinigiano	6	24	3	21	14	1	8	1	13	2
(4) Castell’Azzara	2	4	0	4	2	0	1	1	2	0
(5) Sorano	6	30	6	24	16	3	8	3	16	3
(6) Alberese	1	2	0	2	1	0	1	0	1	0
(7) Maciano	3	11	2	9	5	2	3	1	6	1
(8) Capalbio	2	7	1	6	6	0	0	1	5	1
Sum	22	82	12	70	45	8	21	8	44	9
% on total			14.63	85.37	60.81	10.81	28.38	9.76	83.02	16.98

#### *Vitis vinifera* subsp. *sativa*, *Vitis* Non-*vinifera* and Non-*vitis* Genotypes

Other 77 genotypes were sampled for DNA polymorphism analysis from the vineyard collection of the Department of Agriculture, Food and Environment of University of Pisa (Supplementary Table S1): 70 *Vitis vinifera* subsp. *sativa* genotypes cultivated in Tuscany, including local and international reference varieties introduced in Tuscany more than 50 years ago; ‘Isabel’, a cross between *Vitis vinifera* and the American genotype *Vitis labrusca* (D’Onofrio et al., 2016); two American *Vitis* species used as parents of the most widespread rootstocks (*Vitis riparia* and *Vitis rupestris*) belonging to the subgenus *Euvitis* such as *Vitis vinifera*; a *Vitis rotundifolia* (another American species, but belonging to the subgenus *Muscadinia*); and three *Vitaceae* not belonging to the genus *Vitis* (*Cissus rhombifolia*; *Cayratia japonica;* and *Leea guineensis*) obtained from the Karlsruher Institut für Technologie (KIT) Botanisches Institut.

### Genotyping

#### DNA Extraction

DNA was extracted from young growing leaves (about 2 cm ∅) according to the protocol reported by Mulcahy et al. (1993), and modified as indicated by D’Onofrio et al. (2010).

#### Analysis of Nuclear Microsatellites

The 82 collected putative *Vitis vinifera* subsp. *sylvestris* accessions and the other 77 genotypes were genotyped at 14 nuclear simple sequence repeats (SSRs) loci. To facilitate comparisons with other genotypes in *Vitis* databases, a core set of microsatellites selected from the GrapeGen06 research European Project was included in the analysis: VVS2 (Thomas and Scott, 1993), VVMD5, VVMD7, VVMD28, and VVMD32 (Bowers et al., 1996), VVMD25, VVMD27 (Bowers and Meredith, 1997), VrZAG62 and VrZAG79 (Sefc et al., 1999). The additional five microsatellite loci were: VVMD6 (Bowers et al., 1996), VVMD17, VVMD21, and VVMD24 (Bowers et al., 1999), VMC1b11b (Zyprian and Topfer, 2005).

PCR amplification was conducted in a 20 μl reaction mixture containing 10 ng of genomic DNA, 0.5 U Go Taq Flexi DNA polymerase (Promega, Madison, WI, United States), 1x Go Taq Flexi Colorless PCR buffer (Promega), 1.5 mM MgCl2, 0.5 μM of each primer and 200 μM of each deoxynucleoside triphosphate. A primer of each couple was fluorescently labeled with dye phosphoramidites (6-FAM, HEX, NED, and PET).

PCRs were carried out using a MyCycler Thermal Cycler (Bio-Rad, Hercules, CA, United States), initially set for 4 min at 94°C, followed by 39 cycles of 94°C for 30 s, 55°C for 30 s, and 72°C for 1 min, with a final extension at 72°C for 5 min. The separation and sizing of alleles were performed on an ABI 310 Genetic Analyzer (Applied Biosystems, Weiterstadt, Germany) and analyzed with GeneScan v. 2.1, as reported by D’Onofrio et al. (2016). In each PCR run, ‘Sangiovese’ microsatellites were amplified as a reference to standardize the microsatellite profiles, as implemented in the *Italian Vitis Database* (D’Onofrio and Scalabrelli, 2010)^1^.

#### Analysis of Chloroplast Microsatellites

To determine the chlorotype of the 82 collected putative *Vitis vinifera* subsp. *sylvestris*, 9 chloroplast SSRs were analyzed: ccmp3, ccmp5, and ccmp10 (Weising and Gardner, 1999); ccSSR-5, ccSSR-9 and ccSSR-14 (Chung and Staub, 2003); NTCP-8 (Bryan et al., 1999); ccSSR-23b and VVCP67629 (Péros et al., 2010). PCR amplification was carried out in a 20 μl reaction mixture containing 10 ng of genomic DNA, 0.5 U Go Taq Flexi DNA polymerase (Promega, Madison, WI, United States), 1x Go Taq Flexi Colorless PCR buffer (Promega), 1.5 mM MgCl2, 0.5 μM of each primer and 200 μM of each deoxynucleoside triphosphate. A primer of each couple was fluorescently labeled with a dye phosphoramidites (6-FAM, HEX, NED, and PET).

PCRs were carried out using a MyCycler Thermal Cycler (Bio-Rad, Hercules, CA, United States), initially set for 4 min at 94°C, followed by 35 cycles of 94°C for 1 min, 50°C for 30 s (at 55°C for 30 s for the ccmp5 locus), and 72°C for 1 min, with a final extension at 72°C for 5 min. Allele separation and sizing were performed on a ABI 310 Genetic Analyzer (Applied Biosystems, Weiterstadt, Germany) and analyzed with GeneScan v. 2.1, as reported by D’Onofrio et al. (2016).

#### Analysis of Single Nucleotide Polymorphisms (SNPs)

Of the non-redundant *sylvestris* genotypes, 37 were selected based on the results of the SSRs polymorphism analysis. These *sylvestris* genotypes, along with the other 77 genotypes (Supplementary Table S1) were then subjected to SNPs analysis by the Grapevine Illumina Infinium SNP chip developed by the GrapeReSeq Consortium (GrapeReSeq 18K Vitis genotyping chip), and analyzed by Traits Genetics GmbH (Gatersleben, Germany).

As the dataset includes highly distant genotypes with a high percentage of failed SNPs, the markers were selected using three different procedures: (i) by the missing rate per genotype (<0.1), missingness test (the maximum number of missing SNP states per SNP site, GENO > 0.2) and minor allele frequency (MAF < 0.05) test (matrix SNPs-A) performed by Plink software (Purcell et al., 2007); (ii) or applying the same missingness test and minor allele frequency test including all the genotypes (matrix SNPs-B) even with a high rate of missing markers (>0.1); or (iii) simply selecting only the totally successful SNPs in all the genotypes (matrix SNPs-C).

### Genetic Diversity, Identity, and Genetic Relationships

Redundant *sylvestris* genotypes were identified in base to SSR analysis by CERVUS 3.0.3 (Kalinowski et al., 2007). GenAlEx 6.503 (Peakall and Smouse, 2006, 2012) was used to calculate the following parameters for genetic diversity on the SSRs profile of non-redundant *sylvestris* genotypes, the 70 *Vitis vinifera* subsp. *sativa*, and the 4 *Vitis* non-*vinifera* genotypes: the number of alleles (Na), the effective number of alleles (Ne), observed heterozygosity (Ho), expected heterozygosity (He) (Nei, 1973); and the fixation index (F), also referred to as the inbreeding coefficient.

Allelic richness (AR) and private allelic richness (PAR) for each population were estimated using the rarefaction method, which compensates for differences in sample size (i.e., rarefied allelic richness) among populations as implemented in HP-Rare 1.1 (Kalinowski, 2005). GENEPOP v. 4.7.2 (Rousset, 2008) was used to estimate the effective number of migrants per generation (Nm) among the grapevine populations and between the two subspecies using the private allele method (Barton and Slatkin, 1986), and Weir and Cockerham’s F-statistics (Weir and Cockerham, 1984) per each population (F_*ST*_).

Identity analyses were conducted using CERVUS for all the accessions in the *Italian Vitis Database*^2^ at 9 loci, or at 14 loci for the genotypes uploaded by the Department of Agriculture, Food and Agro-environmental, University of Pisa, and at 9 loci in the *European Vitis Database*^3^.

A preliminary analysis of parent-offspring relationships was conducted among the non-redundant *sylvestris* genotypes, the seventy *Vitis vinifera* subsp. *sativa* and the four *Vitis* non-*vinifera* genotypes, by identity-by-state (IBS) on nuclear SSRs using CERVUS. The parent-offspring, sibling or equivalent relationships were therefore checked on the SNPs-A data set by the identity-by-descent (IBD) coefficients calculated using PLINK (Purcell et al., 2007). The two IBD coefficients inferring the probabilities that a pair shares one or two alleles IBD (Z1, Z2, respectively), and relatedness value (PI_HAT) were calculated for all pair-wise comparisons. The expected values of Z1, Z2, and PI_HAT were, respectively, 1, 0, and 0.5 for parent-offspring (PO); 0.5, 0.25, and 0.5 for full-siblings (FS); and 0.5, 0, and 0.25 for second-degree relatives. The values for known pedigree relationships included in the set of 70 *sativa* genotypes were used to evaluate IBD and relatedness coefficients, thus establishing threshold values.

### Cluster Analysis and Population Structure

The allele sharing distance between each pair of individuals was calculated using PEAS software (Xu et al., 2010) and UPGMA clustering (Sokal and Michener, 1958) performed using MEGA 6 (Tamura et al., 2013). Poppr 2.8.3 (Kamvar et al., 2015) implemented in R 3.6.1 software (R Core Team, 2019) was also used to calculate neighbor-joining unrooted phylogenetic trees based on the Nei distance (Nei, 1972), which was then circle plotted by MEGA 6.

A Bayesian clustering was derived on both nuclear SSRs and SNPs using STRUCTURE (Pritchard et al., 2000) in its revised 2.3.4 version (Jul 2012). An admixture model and independent allelic frequencies were used to analyze the dataset without prior population information. To estimate the cluster number (*K*) of ancestral genetic groups and the ancestry membership proportion of each individual in these clusters, the algorithm was run 10 times for each *K* value from 1 to 10. The optimum number of clusters (*K*) was chosen based on the second order rate of change in the log probability of data between successive *K* values (Evanno et al., 2005).

## Results

### Flower and Berry Phenotype

Of the collected accessions 28.38% had male flowers, 71.62 had female-hermaphrodite flowers, while 9.76% were not classified. Among the female-hermaphrodites, 83.02% had black berries and 16.98% had white berries (Table 1).

### SSRs Polymorphism and Identity Analysis

The nuclear SSRs profiles and the chlorotypes are reported in Supplementary Table S1: the SSRs analysis failed for the three non-*Vitis* genotypes.

Of the 82 putative *sylvestris* accessions analyzed, 12 (14.63%) were redundant genotypes and were identified in 4 of the 22 populations sampled (Table 1).

The number of nuclear SSR alleles in the 70 putative *Vitis vinifera* subsp. *sylvestris* and the 70 grapevine genotypes cultivated in Tuscany (*Vitis vinifera* subsp. *sativa*) ranged from 6 for VVMD17 to 19 for VVMD28 and VMC1b11, with a mean value of 13.143 alleles per locus (Table 2). The number of effective alleles ranged from 2.318 for VVMD21 to 7.849 for VVMD28, and the mean overall value was 4.988. The He ranged from 0.569 for VVMD21 to 0.873 for VVMD28, with an average of 0.781. The Ho ranged from 0.486 (VVMD21) to 0.854 (VVS2) and the mean value was 0.726. The mean *F* value for the dataset was 0.073, with the lowest of −0.015 for VVS2 and the highest of 0.180 for VVMD17.

**TABLE 2:** Nuclear SSRs genetic diversity indices of 70 putative *Vitis vinifera* subsp. *sylvestris* and 70 cultivated grapevine (*Vitis vinifera* subsp. *sativa*) genotypes from Tuscany.

Locus	**Na**	**Ne**	**He**	**Ho**	***F***
VVS2	15	6.323	0.842	0.854	−0.015
VVMD5	16	5.534	0.819	0.722	0.118
VVMD7	14	5.887	0.830	0.771	0.071
VVMD27	16	5.722	0.825	0.785	0.049
VrZAG62	11	4.115	0.757	0.688	0.092
VrZAG79	13	4.958	0.798	0.785	0.017
VVMD25	11	4.510	0.778	0.764	0.018
VVMD28	19	7.849	0.873	0.785	0.101
VVMD32	15	5.557	0.820	0.778	0.052
VVMD6	9	4.000	0.750	0.688	0.083
VVMD17	6	3.150	0.683	0.559	0.180
VVMD21	10	2.318	0.569	0.486	0.145
VVMD24	10	4.102	0.756	0.706	0.066
VMC1b11	19	5.807	0.828	0.792	0.044
Mean	13.143	4.988	0.781	0.726	0.073

*Na, no. of different alleles; Ne, no. of effective alleles; Ho, observed heterozygosity; He, expected heterozygosity; F, fixation index.*

Concerning the genetic diversity among populations (Table 3A), the number of alleles per locus (Na) was identical in both *sativa* and *sylvestris* populations (9.714), but lower for the non-*vinifera* population (5.643). On the other hand, the number of effective alleles per locus (Ne) was higher in the *sativa* population (5.040) than in *sylvestris* (3.789), and the value of the non-*vinifera* population was in the middle (4.789). There was a similar trend for observed and expected heterozygosity. The allelic richness in *sativa* was higher than in *sylvestris*, while the private allele richness was higher in *sylvestris*: *sylvestris* genotypes had 10 private alleles in 6 of the 14 SSRs loci analyzed which could be useful in distinguishing *sylvestris* from *sativa* genotypes. The mean Shannon Information Index (I) for the wild accessions (1.581) was lower than for *sativa* genotypes (1.775) and the non-*vinifera* population (1.603). The inbreeding coefficient within individuals (F) was negative for *sativa* (−0.016) and positive for *sylvestris* (0.075) and non-*vinifera* genotypes (0.130).

**TABLE 3:** **(A)** Nuclear SSRs genetic diversity estimates for each analyzed population: 70 *Vitis vinifera* subsp. *sativa* (Sat), 70 *Vitis vinifera* subsp. *sylvestris* (AllSyl), 4 non-*vinifera* (NoVin). **(B)** Then, the analysis war repeated including only the 61 true-*sylvestris* genotypes (TrueSyl).

	**Na**	**Ne**	**AR**	**PAR**	**I**	**Ho**	**He**	**F**
**(A) With all putative sylvestris genotypes (AllSyl)**								
Sat	9.714	5.040	3.770	1.240	1.775	0.791	0.777	−0.016
AllSyl	9.714	3.789	3.560	1.340	1.581	0.663	0.711	0.075
NoVin	5.643	4.789	4.590	2.910	1.603	0.667	0.765	0.130
**(B) With only true-sylvestris genotypes (TrueSyl)**								
Sat	9.714	5.040	3.770	1.260	1.775	0.791	0.777	−0.016
TrueSyl	8.571	3.510	3.550	1.340	1.500	0.646	0.689	0.071
NoVin	5.643	4.789	4.590	2.900	1.603	0.667	0.765	0.130

*Na, no. of different alleles; Ne, no. of effective alleles; AR, allelic richness; PAR, private allele richness; I, Shannon’s information index; Ho, observed heterozygosity; He, expected heterozygosity; F, fixation index.*

The heterozygosity (Ho) was also lower in *sylvestris* than the *sativa* population (0.663 and 0.791, respectively) as was the expected heterozygosity (He) (0.711 and 0.777, respectively). The value of Ne, I, Ho, He, and F were in agreement with previously observed values in *sylvestris* from central Italy (Biagini et al., 2014).

The effective number of migrants per generation (Nm) after correction for sample size was 1.133 (data not shown) among the three populations (*sativa*, *sylvestris*, non-*vinifera*). On the other hand between the *sativa* and *sylvestris* populations, it was 3.224, and again much lower (0.379) between *sativa* and non-*vinifera* and between *sylvestris* and non-*vinifera* (0.444) populations (Table 4A). The Weir and Cockerham F-statistics (Weir and Cockerham, 1984) F_*ST*_ between *sativa* and *sylvestris* populations was 0.067, while between *sativa* and non-*vinifera*, it was 0.106 and between *sylvestris* and non-*vinifera* populations, 0.148 (Table 4A).

**TABLE 4:** Effective number of migrants per generation (Nm) and Weir and Cockerham’s F-statistics among the grapevine populations. **(A)** 70 *Vitis vinifera* subsp. *sativa* (Sat), 70 *Vitis vinifera* subsp. *sylvestris* (AllSyl), 4 non-*vinifera* (NoVin); **(B)** 70 *Vitis vinifera* subsp. *sativa* (Sat), 61 true-*sylvestris* genotypes (TrueSyl), 4 non-*vinifera* (NoVin).

**Population**	**Nm**		**Fst**	
	**Sat**	**NoVin**	**Sat**	**NoVin**
**(A) with all putative sylvestris genotypes (AllSyl)**				
AllSyl	3.224	0.444	0.067	0.148
NoVin	0.379		0.106	
**(B) with only true-sylvestris genotypes (TrueSyl)**				
TrueSyl	2.964	0.294	0.082	0.182
NoVin	0.379		0.106	

*Nm, effective number of migrants per generation; Fst, Weir and Cockerham’s F-statistics.*

The analysis of chloroplast microsatellites revealed three chlorotypes: A, B and D, as indicated by Arroyo-García et al. (2006) (Supplementary Table S1). In agreement with previous studies (Arroyo-García et al., 2006; Grassi et al., 2006; Cunha et al., 2009; Butorac et al., 2018), the majority of the identified genotypes of putative *sylvestris* had chlorotype A (51 samples; 72.86%), while 18 samples (25.71%) had chlorotype D as in the majority of the *Vitis vinifera* subsp. *sativa* cultivated in Italy. Only one genotype (1.43%) had chlorotype B, which has not previously been identified in any Italian *sylvestris*, but only in a few Italian cultivated varieties (Arroyo-García et al., 2006).

### Single Nucleotide Polymorphisms

**FIGURE 2:**
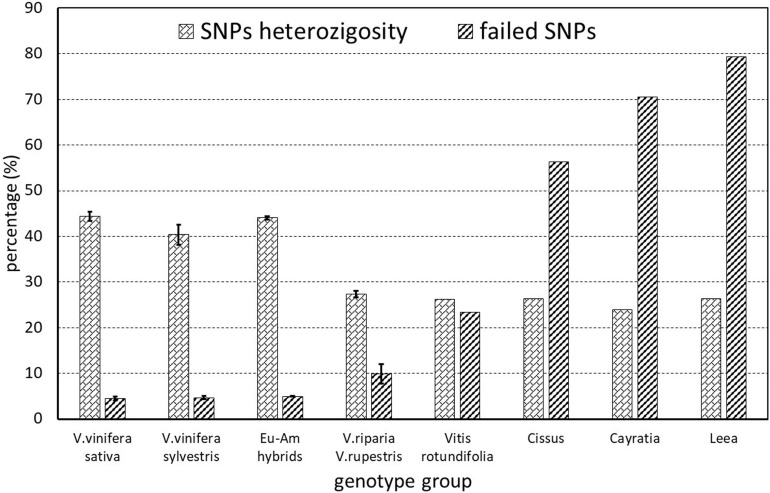
SNPs missing rate and heterozygosity of the various genetic groups analyzed. The European–American groups (Eu-Am hybrids) include the hybrid ‘Isabel’ and the hybrid ‘Seibel 128’ identified among the putative *sylvestris*.

Of the 18071 SNPs in the array, 623 (3.4%) completely failed and were thus removed from the matrix when calculating the subsequent statistics. The percentage of other failed markers ranged from 0.57 to 78.54%. It was mainly related to the taxonomic groups, increasing gradually with the genetic distance from *Vitis vinifera*, and was similar among subsp. *sativa* and putative subsp. *sylvestris* genotypes (Figure 2). However, the heterozygosity ranged from 23.9 to 47.15%, in an inverse trend to percentage of failed markers, and was lower in *sylvestris* (36.90–45.20) than in *sativa* (41.91–47.15) genotypes (Supplementary Table S2). Among the *sativa*, the French genotypes typically have a slightly higher level of heterozygosity than other cultivated genotypes, particularly ‘Sauvignon blanc’ and ‘Pinot noir’ (Supplementary Table S2).

The SNP quality check excluded four genotypes with a missing rate over 10% (*Vitis rotundifolia* and the three non-*Vitis* genotypes) and revealed that 267 SNPs failed the missingness test (the maximum number of missing SNP states per SNP site, GENO > 0.2) and 4751 SNPs failed the minor allele frequency test (MAF < 0.05). Therefore, after frequency and genotyping pruning, there were 13154 SNPs in the dataset (SNPs-A).

Including all the 114 genotypes, even together with the *Leea guineensis* with a missing rate of 78.54%, 313 SNPs failed the missingness test (the maximum number of missing SNP states per SNP site, GENO > 0.2) and 4792 SNPs failed the minor allele frequency test (MAF < 0.05), and 13080 SNPs passed the tests (SNPs-B). Finally, a third matrix (SNPs-C) included only the SNPs (1070) that were successful in the whole set of 114 genotypes (Supplementary Table S3).

### Identity and Parentage Analysis

The identity analysis using the SSRs profiles of genotypes in the *Italian Vitis Database*^4^ and the *European Vitis Database*^5^ indicated that three accessions matched with previously characterized genotypes (Supplementary Table S1).

The genotype Syl_55, identified in the Sorano area (Sorano1), which was hermaphrodite and had white berries, was the Italian variety ‘Bellone’ (Bellone ITA388-G012 EVD; *European Vitis Database*). The Syl_60 recovered from the same population (Sorano1) matched the table grapevine ‘Regina dei vigneti’ (FRA139-1078Mtp1, *European Vitis Database*), and again being hermaphrodite with white berries confirmed this.

Another genotype Syl_3 was identified at Campiglia D’Orcia, in a flat area near a stream named Formone, which was hermaphrodite and had black berries, and matched the European-American *Vitis* interspecific crossing ‘Seibel 128’ (Seibel 128 FRA139-5026Mtp4, *European Vitis Database*).

The parent-offspring analysis performed by identity-by-state (IBS) on nuclear SSRs and by identity-by-descent (IBD) on the SNP profile indicated that some accessions were possibly natural *sativa*-*sylvestris* crosses, while others might be crosses between *sylvestris* (Supplementary Table S1). The IBD analysis was performed on the SNPs-A data set with a threshold of 0.462 PI_HAT identified as the lowest pair-wise relationship (Cabernet sauvignon-Sauvignon blanc) among the known PO relationships in the set of the 70 *sativa* genotypes.

Of the 67 non-redundant genotypes not matching known genotypes, six (8.95%) appeared to be parent-offspring of grapevine varieties cultivated in Tuscany (Supplementary Table S1).

The accession Syl_26 identified in the area of Cinigiano at Poggi del Sasso, which was hermaphrodite with black berries, appeared to be a natural cross of ‘Sangiovese’. Another natural cross of ‘Sangiovese’ was the accession Syl_16. This genotype was identified in a population in the area of Manciano and had hermaphrodite flowers and black berries. The genotype Syl_10, identified at Castell’Azzara, which was female and had black berries, appeared to be a natural cross of ‘Trebbiano toscano.’ Again, this relationship was confirmed by both SSRs and SNPs. The Syl_69, from the Sorano area, which had female flowers and produces black grapes, was a cross with another genotype of the same population, the Syl_55 identified as ‘Bellone.’ Another genotype (Syl_56) from the same area (Sorano) was a natural cross of the hybrid ‘Isabel’. This accession had female flowers and produces black grapes.

From an analysis exclusively on nuclear SSRs, the genotype Syl_27, with female flowers and white berries from Cinigiano, was identified as a natural cross of ‘Berzemino/Negretta toscana’, a local Tuscany variety reported in the Italian Vitis Database^6^.

The analysis of only the SSRs profile, and not SNPs because of the lack of profiles, indicated a parent-offspring relationship between the *sylvestris* genotypes Syl_49 and Syl_63 from Sorano, and between Syl_54 and Syl_61 also from Sorano (Supplementary Table S1).

In addition, the IBD analysis suggested a full-sibling relationship between Syl_4 and Syl_7 from Capalbio, and between Syl_48 and Syl_49 from Sorano area (Supplementary Table S1).

The second-degree relationship between Syl_26 and Syl_16, as well as their second-degree relationship with ‘Ciliegiolo,’ a well-known offspring of ‘Sangiovese,’ confirmed that both these accessions were ‘Sangiovese’ crosses. In addition, the second-degree relationship between Syl_10 and ‘San Lorenzo’ (Sat_4), a local Tuscan offspring of the ‘Trebbiano toscano’ variety, confirmed this parent-offspring relation with ‘Trebbiano toscano.’

Interestingly, the IBD analysis of highlighted some second-degree relationships among ‘Petit manseng’ (Sat_38), ‘Petit verdot’ (Sat_48), ‘Greco bianco’ (Sat_7) and some *sylvestris*.

### Cluster Analysis

**FIGURE 3:**
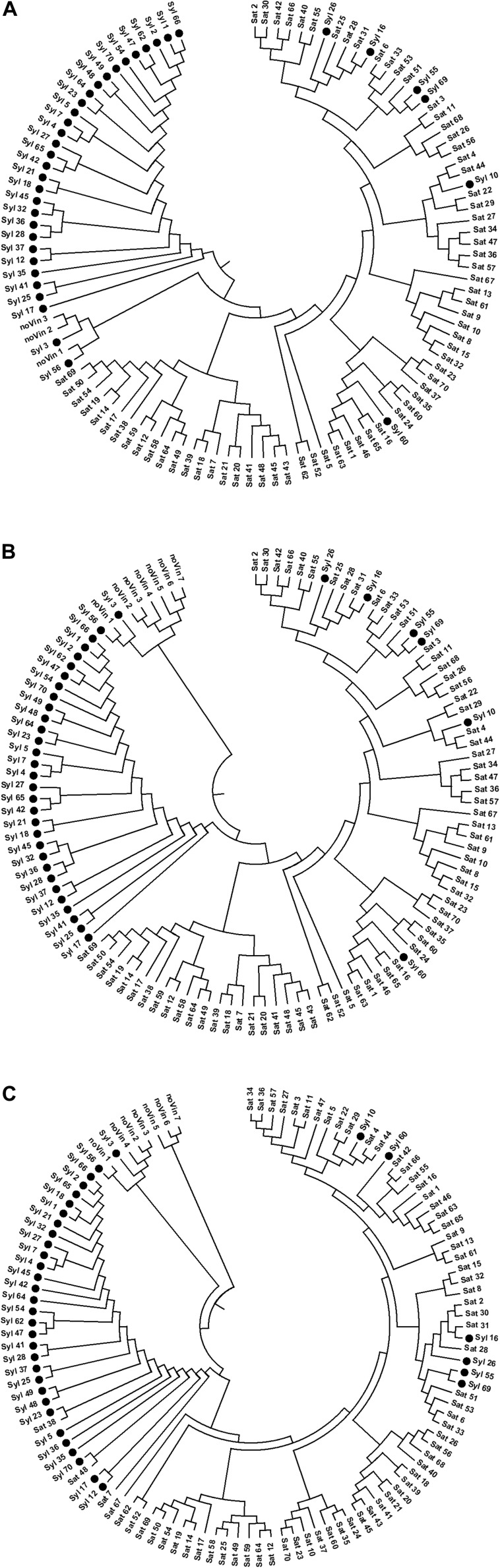
UPGMA-trees performed on SNPs. **(A)** 13154 SNPs; **(B)** 13080 SNPs; **(C)** 1070 SNPs.

Three UPGMA tree-A, tree-B, and tree-C, were generated by the allele sharing distance between each pair of individuals on selected SNPs in SNPs-A, SNPs-B, and SNPs-C matrices, respectively (Figure 3), using the PEAS and MEGA packages.

In all UPGMA trees there was a clear separation of three main clusters: a cluster with non-*vinifera* genotypes, another with the majority of *sylvestris* genotypes, and the last including almost all *sativa* genotypes, non-true-*sylvestris* genotypes and putative *sylvestris*-*sativa* crosses.

In the tree-A obtained from the matrix of the selected SNPs through the missingness and minor allele frequency test on genotypes with a low rate of missing markers (<10%) and without *Vitis rotundifola* and non-*vitis* genotypes, the non-*vinifera* cluster was between the *sylvestris* and *sativa* clusters. While, in tree-B, which was made up of the matrices including the *Vitis rotundifola* and non-*vitis* genotypes with a high missing rate (over 10%), the cluster of *sylvestris* genotypes was between the non-*vinifera* and *sativa* clusters. In both these trees, the arrangement of genotypes within *sylvestris* and *sativa* clusters was exactly the same. In the *sativa* cluster, there are two main sub-clusters: one including most of the Italian *sativa* and Muscat varieties, and another grouping most of the French genotypes and some Italian genotypes. In tree-C, although generated from a low number of SNPs (1070), the cluster of *sylvestris* was between the non-*vinifera* and *sativa* genotypes, but the arrangement of genotypes within *sylvestris* and *sativa* clusters has some differences.

In all trees, the *sylvestris* accessions identified as the European–American *Vitis* hybrid ‘Seibel 128’ (Syl_3), and the accession identified as a full-sibling of the European-American *Vitis* hybrid ‘Isabel’ (Syl_56) were in the non-*vinifera* cluster. In addition, the accessions Syl_55 and Syl_60, matching the cultivated varieties ‘Bellone’ and ‘Regina dei vigneti,’ respectively, were properly in the *sativa* cluster.

The Syl_26 and Syl_16 were always in the sub-cluster with ‘Sangiovese,’ confirming these accessions as crosses of ‘Sangiovese’ (Sat_31), the most widely diffused grapevine in Tuscany. Similarly, the Syl_10 was in the sub-cluster including ‘Trebbiano toscano’ (Sat_44), confirming its origin as a cross of this important variety that is widespread in Tuscany. In addition, Syl_69 was among the *sativa* genotypes, near Syl_55 identified as ‘Bellone,’ thus confirming the parent-offspring relationship between these two genotypes.

In tree-C, the non-*vitis* genotypes were rightly in the non-*vinifera* cluster, and unlike the other two trees, the *sativa* genotypes Sat_38, Sat_48 and Sat_7 (respectively ‘Petit Manseng,’ ‘Petit Verdot’ and ‘Greco bianco’) were in the cluster of *sylvestris* genotypes, in agreement with the IBD second-degree relationships.

**FIGURE 4:**
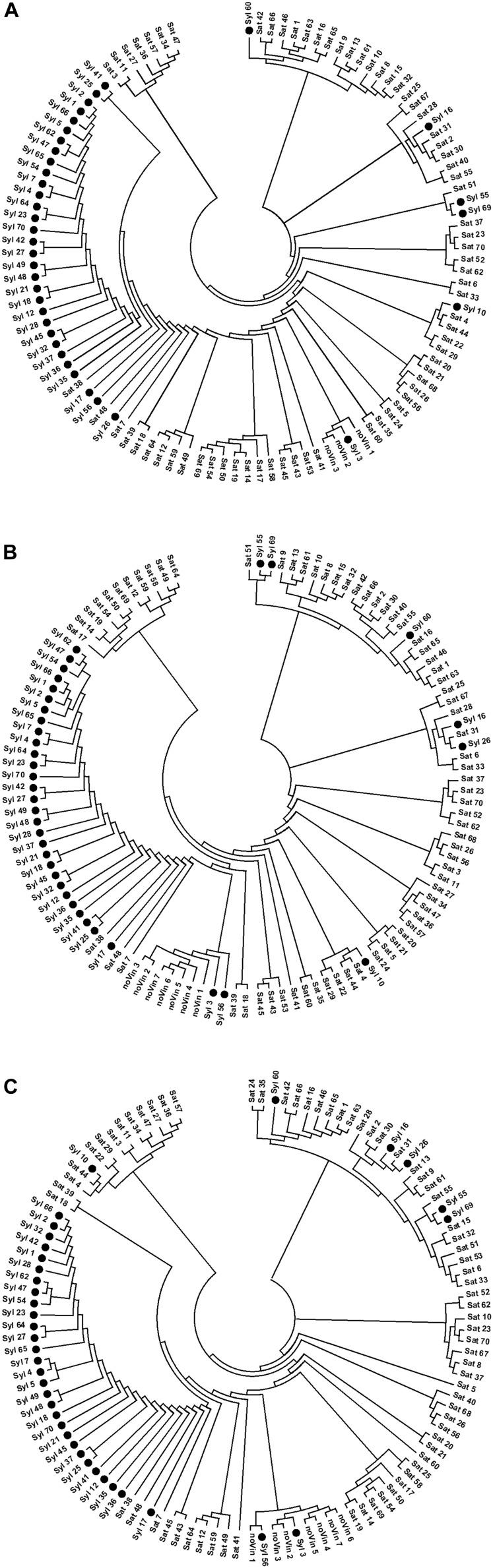
NJ-dendrograms performed on SNPs. **(A)** 13154 SNPs; **(B)** 13080 SNPs; **(C)** 1070 SNPs.

Similarly to the UPGMA trees, three unrooted NJ-dendrograms (NJ-A, NJ-B, and NJ-C, respectively) were generated from SNPs-A, SNPs-B, and SNPs-C matrices by neighbor-joining (NJ) cluster analysis based on the pair-wise Nei’s distance (Figure 4). All three NJ-dendrograms clearly differentiated the three main clusters: *sylvestris* cluster, *sativa* cluster, and the cluster with non-*vinifera* genotypes. The cluster of non-*vinifera* accessions was among the *sativa* and *sylvestris* clusters in the NJ-B dendrogram but among *sativa* genotypes in NJ-A and NJ-C dendrograms.

In agreement with the UPGMA trees, in all the NJ-dendrograms, the two naturalized *sativa* (Syl_55 and Syl_60) and the four sativa-*sylvestris* (Syl_10, Syl_16, Syl_26, and Syl_69) crosses were all in the *sativa* cluster. In addition, the non-true *sylvestris* accessions Syl_3 and Syl_56 related to the hybrid ‘Isabel’ were in the non-*vinifera* cluster in NJ-B and NJ-C dendrograms as in all UPGMA trees, while in NJ-A, Syl_3 was confirmed in the non-*vinifera* cluster but Syl_56 appeared in the *sylvestris* cluster. In all NJ dendrograms, the *sativa* genotypes ‘Petit manseng’ (Sat_38), ‘Petit verdot’ (Sat_48) and ‘Greco bianco’ (Sat_7) were in the sylvestris cluster, as in the UPGMA tree-C and in agreement with the IBD second-degree relationships.

### Analysis of Ancestry

**FIGURE 5:**
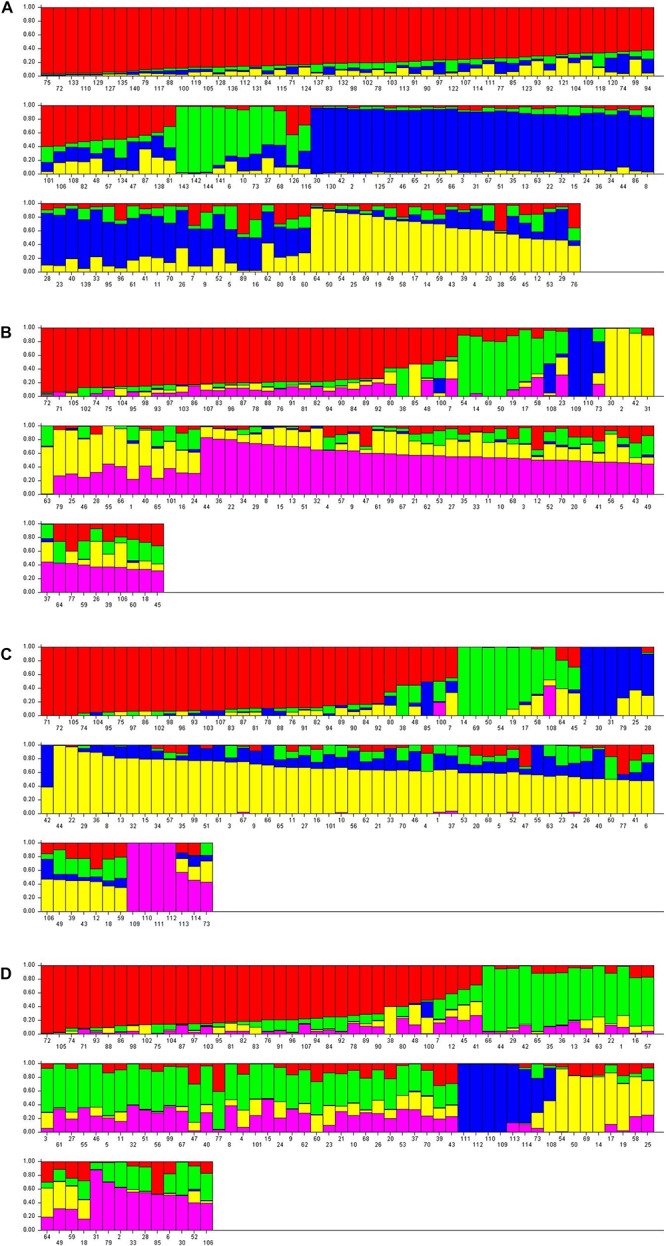
Barplot displaying the admixture proportions of wild, cultivated grapevine and non-*vinifera* genotypes as estimated by STRUCTURE analysis. **(A)** 144 genotypes, 14 SSR loci, *K* = 4; **(B)** 110 genotypes, 13154 SNPs, *K* = 5; **(C)** 114 genotypes, 13080 SNPs, *K* = 5; **(D)** 114 genotypes, 1070 SNPs, *K* = 5.

The structure analysis was performed on both nuclear SSR markers (barplot-A) and SNP matrices (barplot-B, barplot-C, barplot-D from SNPs-A, SNPs-B, SNPs-C matrices, respectively) (Figure 5).

Analysis on the SSRs of 144 genotypes (70 *sativa*, 70 putative *sylvestris*, three non-*vinifera*, *Vitis rotundifolia*, but not the three non-*Vitis* genotypes because the SSRs failed) revealed four populations (Figure 5A): non-*vinifera* genotypes, true-*sylvestris*, most Italian *sativa* and Muscat varieties, and most French *sativa* and some Italian *sativa* varieties (Figure 5). In agreement with the cluster analysis, the Italian *sativa* population included the two naturalized *sativa* (Syl_55 and Syl_60; accessions 125 and 130, respectively) and the four *sativa-sylvestris* crosses (Syl_10, Syl_16, Syl_26 and Syl_69; accessions 80, 86, 96, and 139, respectively), but also the genotype Syl_19 (accession 89) not analyzed by SNPs. Another *sylvestris* genotype (Syl_6; accession 76), not analyzed by SNPs, was in the French population, and another (Syl_46; accession 116) was in the cluster of non-*vinifera* genotypes. The presence of ‘Petit verdot’ (Sat_48; accession 48) in the population of *sylvestris* was also confirmed, but not the varieties ‘Petit manseng’ (Sat_38; accession 38) and ‘Greco bianco’ (Sat_7; accession 7), which were respectively assigned to the French *sativa* and Italian *sativa* cluster. In addition, the local Tuscan varieties ‘Colorino’ (Sat_47; accession 47) and ‘Schiava gentile’ (Sat_57; accession 57) appeared among *sylvestris*.

The structure analysis on all the three SNP matrices always revealed five populations: non-*vinifera*, *sylvestris*, Italian *sativa*, French *sativa*, and a population including all Muscat varieties, the ‘Sangiovese’ and a few other Tuscan varieties. The barplot-B and C, from SNP-A and SNPs-B matrices, appeared very similar (Figures 5B,C), and in agreement with UPGMA tree-C and all NJ dendrograms, the varieties ‘Petit manseng’ (Sat_38; accession 38), ‘Petit verdot’ (Syl_48, accession 48) and Greco bianco’ (Sat_7; accession 7) were included in the *sylvestris* population. The naturalized *sativa* genotypes (Syl_55 and Syl_60; accessions 99 and 101, respectively) and most of the *sativa* offspring were in the *sativa* population, as well as the naturalized hybrid ‘Seibel 128’ (Syl_3; accession 73), and the full-siblings of the hybrid ‘Isabel’ (Syl_56; accession 100) were in the non-*vinifera* population. Looking at the ancestry of the single genotypes, it seems that barplot-C better separated the *sylvestris* and *sativa* ancestries.

The barplot-D generated from the matrix SNPc-C including the non-*vitis* genotypes but with only the 1070 SNPs that were successful in all genotypes, confirmed the same 5 populations (Figure 5D). All the non-*vinifera* and non-*vitis* genotypes were in the same cluster together the naturalized hybrid ‘Seibel 128’ (Syl_3; accession 73), while the descendant of the hybrid ‘Isabel’ (Syl_56; accession 100) was in the *sativa* population despite a high non-*vinifera* ancestry. In addition to ‘Petit manseng’ (Sat_38; accession 38), ‘Petit verdot’ (Syl_48, accession 48) and ‘Greco bianco’ (Sat_7; accession 7), also the *sativa* varieties ‘Cabernet franc’ (Sat_12; accession 12), ‘Syrah (Sat_45; accession 45) and ‘Roussane bianco’ (Sat_41; accession 41) were in the *sylvestris* population although their *sylvestris* ancestry is less than 50%.

## Discussion

The genotyping analysis of the nuclear and chloroplast SSRs and SNPs polymorphism of putative wild vines identified and recovered in southern Tuscany highlights the difficulty of identifying true *Vitis vinifera* subsp. *sylvestris*.

The presence of redundant genotypes and their high percentage in 6 out of 22 sampled populations, and the identification of redundant plants over 20 m from each other, suggest not only the possible extent of such plants, but also the possibility of natural vegetative propagation.

The identity analysis of the main *Vitis* databases revealed that 3 of the 70 non-redundant putative wild genotypes are known genotypes: two are *Vitis vinifera* subsp. *sativa* varieties (‘Bellone’ and ‘Regina dei vigneti’) and one is the European-American *Vitis* hybrid ‘Seibel 128.’ Two of these (‘Bellone’ and ‘Regina dei vigneti’) were collected from the same population in the Sorano area, while the other (‘Seibel 128’) was identified in a population about 20 km away (Castiglione D’Orcia). This locality is not currently an intensive wine viticulture area, but the known naturalized genotypes identified suggest that this was previously a cultivation area of allochthonous grapevine varieties, which was later abandoned, and consequently the cultivated grapevine genotypes acquired a naturalized appearance, as previously also observed for other *sativa* genotypes around Europe (Riaz et al., 2018). Their identity is supported by the high level of *sativa* ancestry of the genotypes identified as ‘Bellone’ and ‘Regina dei vigneti’ (Supplementary Table S2), and their position in the cluster of *sativa* in all the UPGMA-trees and NJ-dendrograms. Other evidence includes the high non-*vinifera* ancestry of the genotypes identified as ‘Seibel 128’ and their position in the cluster of non-*vinifera* in all the UPGMA-trees and NJ-dendrograms.

Six (8.95%) of the 67 putative *sylvetris* genotypes studied appeared to be natural crosses of the most widespread grapevine varieties in Tuscany (‘Sangiovese’ and ‘Trebbiano toscano’), of a minor local variety (‘Berzemino’), of the non-true *sylvestris* matching ‘Bellone,’ and of the European-American *Vitis* hybrid ‘Isabel.’ These results suggest a previous pollen flow between wild and cultivated grapevine proven by the analysis of seeds collected from *sylvestris* grapevines (Di Vecchi-Staraz et al., 2008). Four of the six crosses observed had chlorotype A, like most of the *sylvestris* genotypes, and only one had chlorotype D, like most cultivated *sativa* varieties in Italy. The progeny usually inherits the mother’s chlorotype, thus the higher number of crosses with chlorotype A suggest a higher pollen flow from *sativa* to the *sylvestris* genotypes compared to the opposite direction. This is also supported by the probable higher quantity of *sativa* than *sylvetris* pollen in highly intensive viticulture regions such as Tuscany. However, seedlings from wild plants have a higher probability of growing in woods than those from cultivated plants in areas with worked soil. The two pairs of *sylvestris* genotypes with a parent-offspring relationship (Syl_49-Syl_63, Syl_54-Syl_61) and the two pairs with a full-sibling relationship (Syl_4-Syl_7, Syl_48-Syl_49) had chlorotype A and a high *sylvestris* ancestry, confirming their true *sylvestris* nature.

All the above described parent-offspring and full-siblings highlight sexual reproductive activities among *sylvestris* and *sylvestris*-*sativa* genotypes.

The population of *sativa* had a higher number of SSRs effective alleles and allele richness than the *sylvestris* population (Table 3A), highlighting a higher diversity among *sativa* genotypes cultivated in Tuscany, coming from western to eastern Europe, compared to *sylvestris* genotypes collected from a few isolated populations in a relative small area (about 100 km^2^) in southern Tuscany. These differences increased with the exclusion from the *sylvestris* population of accessions that were non-true-*sylvestris* according to the parentage analysis (Table 3B).

The expected and observed heterozygosity calculated on SSR markers appeared higher in *sativa* than in *sylvestris* population, in agreement with previous results (Riaz et al., 2018). In addition, the Ho value of the *sativa* population appeared to be slightly higher than the He values, while the reverse was true for the *sylvestris* accessions. These differences correspond with the positive *F* values in *sylvestris*, which suggests a high level of genetic relationship among the individuals from the same wild populations (Table 3).

With the exclusion from the *sylvestris* population of accessions that were non-true-*sylvestris* the F_*ST*_ between *sativa* and *sylvestris* populations increased, while the effective number of migrants per generation decreased (Table 4). The F_*ST*_ indicated that the overall level of genetic differentiation between *sativa* and *sylvestris* genotypes was moderate, in agreement with previous results (Marrano et al., 2018; Riaz et al., 2018). However it was higher between *sativa* and non-*vinifera* genotypes and even higher between *sylvestris* and non-*vinifera* genotypes (Table 4B).

All these results are in agreement with previous studies attributing the low level of diversity among *sylvestris* and the absence of an inter-*sylvestris* population gene-flow to the small size and the isolation of the *sylvestris* populations, and the high level of diversity within cultivated genotypes to the sexual crossing occurring during the grapevine domestication (This et al., 2006; Di Vecchi-Staraz et al., 2008; Vitti et al., 2013; Marrano et al., 2017; Riaz et al., 2018).

Also the SNPs heterozygosity was higher in the *sativa* grapevine compared to *sylvestris*, supporting previous observations based on SNPs analysis (Lijavetzky et al., 2007; Emanuelli et al., 2013; Marrano et al., 2017). With the exclusion of the accessions indicated as non-true-*sylvestris*, the SNPs heterozygosity of *sylvestris* further decreased to a range of 37–41% (Supplementary Table S2). Thus, the heterozygosity range of true-*sylvestris* appears to be up to 6% less and does not overlap with that of *sativa* genotypes. Consequently, the SNPs heterozygosity revealed by the GrapeReSeq 18K Vitis genotyping chip could be a useful additional method for separating *sylvestris* and *sativa* genotypes, together other SNPs genomic signatures that are able to differentiate between these two sub-taxa (Marrano et al., 2018).

Therefore, although a higher level of heterozygosity is expected in *sylvestris* because of its obligate out-crossing nature compared to *sativa*, the results indicate a lower level of diversity in *sylvestris* than *sativa*, and a high level of genetic relationship among individuals from the same wild populations. This is in agreement with other studies which highlight that man-made and natural geographical barriers can also lead to the isolation of wild populations in their native habitat, and could lead to significant inbreeding, reduced gene flow within and among different geographic groups (Bacilieri et al., 2013; Emanuelli et al., 2013; Imazio et al., 2013; Riaz et al., 2018).

The UPGMA-trees and structure barplots from SNPs were generally more accurate than the SNPs NJ-dendrograms in the separation of populations. All the UPGMA-trees and barplots from SNPs clearly separate the true *sylvestris* genotypes from *sativa* and non-*vinifera* genotypes, while in the NJ-dendrograms, the cluster of non-*vinifera* genotypes is mainly among *sativa* genotypes. Among the sativa population, the UPGMA-trees clearly separate French genotypes (representing the *proles occidentalis*) from Italian and Muscat genotypes. The barplot from the structure analysis also appeared more informative than the UPGMA-trees because it further separated the Italian *sativa* (representing the *proles pontica*) from Muscat genotypes (representing the *proles orientalis* subpr. *caspica*), in agreement with Marrano et al. (2015). The UPGMA-tree and barplot obtained from the SNPs-B matrix of 13080 SNPs selected by the missingness (GENO > 0.2) and minor allele frequency (MAF < 0.05) tests including the non-*vinifera* genotypes with a high rate of failed SNPs (>10%), appeared to be slightly more informative and probable than the tree generated by the matrix of the 13154 SNPs selected only on genotypes with a low rate of failed SNPs (<10%).

With regard to the single genotypes, all the UPGMA-trees, NJ-dendrograms and barplot, correctly assigned the naturalized *sativa* (Syl_55 and Syl_60), as well as the naturalized European-American *Vitis* interspecific hybrid ‘Seibel 128’ expectedly in the non-*vinifera* cluster. The UPGMA-tree generated by the matrix SNPs-C, including only the 1070 SNPs that were successful in all 114 genotypes genotyped by SNPs, although generally less accurate, placed the French varieties ‘Petit Manseng’ and ‘Petit Verdot’ and the Italian variety ‘Greco bianco’ among sylvestris. This was in agreement with all the NJ-dendrograms, all the barplots from SNPs, and also in agreement with the evidence of a second-degree relationship of these genotypes with sylvestris as indicated by IBD analysis. In addition, in the barplot generated by the SNPs-C matrix also ‘Cabernet franc’, ‘Syrah and ‘Roussane bianco’ are among the sylvestris.

The barplot from SSRs (Figure 5A) appears to be highly informative despite the low number of markers (14 loci) compared to the high number of SNPs, although it is less accurate in the separation of genotypes at the edges of the populations. Although it correctly separates the true *sylvestris* from naturalized *sativa* and hybrids, only ‘Petit verdot’ is in the cluster of *sylvestris*, while ‘Petit manseng’ and ‘Greco bianco’ are among the *sativa* genotypes, even though showing a visible *sylvestris* ancestry in the barplot. However the Tuscan *sativa* varieties ‘Colorino’ and ‘Schiava gentile’ are assigned to the population of *sylvestris*.

These results suggest an introgression of *sylvestris* into important cultivated *sativa* varieties, supporting the previous hypothesis that in the areas of grapevine cultivation, the wild germplasm contributed to creating some of the current cultivated varieties (De Andrés et al., 2012; Riaz et al., 2018). The high *sylvestris* ancestry in ‘Petit Verdot’ has been previously highlighted, together with another five French (Arvine Petite, Cot, Chenin Blanc, Pinot Meunier and Sauvignon Blanc) and five Spanish (Albariño, Caiño Blanco, Ferrón, Maturana, Ondarrabi Betlza) varieties. This research adds the ‘Petit manseng’ to this group of varieties, and possibly also ‘Cabernet franc’ and ‘Syrah, strengthening the hypothesis of a second center of domestication of cultivated grape in Western Europe, in addition to the primary center of domestication in Transcaucasia (Riaz et al., 2018).

The introgression of *sylvestris* in the Italian cultivated variety ‘Greco bianco,’ but possibly also in the Tuscany local varieties ‘Colorino,’ ‘Schiava gentile’ and ‘Roussane bianco,’ highlights an additional center of domestication in Tuscany.

The identification of a hybrid between a *sylvestris* and the European-American hybrid ‘Isabel’ and a putative *sylvestris* with a high non-*vinifera* ancestry also support a genetic flux among non-*vinifera* and *sylvestris* genotypes. Thus, there is a general flux among the various taxa of grapevine in intensive viticulture areas.

## Conclusion

The results of this research highlight a higher pollen flow from *sativa* to *sylvestris* genotypes than in the opposite direction, and thus a significant introgression of cultivated genotypes into wild grapevines. The evidence of introgression of *sylvestris* genotypes into cultivated varieties, although at a much lower rate than in the opposite direction, also supports previous research suggesting separate domestication events in Western Europe, in addition to the primary ancient center of domestication from the Near East to Central Asia.

It is therefore plausible that crosses among *sylvestris* and *sativa* occurred in the numerous centers of diversification along the migration routes, as well as crosses among *sativa* genotypes, and that these processes are still ongoing despite the reduction in *sylvestris* populations.

Finally, the genotype (Syl_26) identified as a cross between ‘Sangiovese’ and a *sylvestris*, called ‘Femmina Poggi,’ produces grapes of an acceptable quality which are suitable for the production of an interesting wine with a strong black color and intense fruity and spicy notes.

## Data Availability Statement

The datasets generated for this study can be found in NCBI and a full list of urls and accession can be found in Supplementary Table S1.

## Author Contributions

The author confirms being the sole contributor of this work and has approved it for publication.

## Conflict of Interest

The author declares that the research was conducted in the absence of any commercial or financial relationships that could be construed as a potential conflict of interest.

## Footnotes

1 www.vitisdb.it

2 www.vitisdb.it

3 http://www.vitisdb.it/accessions/show/16945

4 www.vitisdb.it

5 http://www.eu-vitis.de

6 http://www.vitisdb.it/accessions/show/16945

## Supplementary Material

TABLE S1 SSRs allelic profile and chlorotype of 70 putative *Vitis vinifera* subsp. *sylvestris* collected in southern Tuscany, 70 *Vitis vinifera* subsp. *sativa*, 3 *Vitis* non-*vinifera* and 4 non-*Vitis* genotypes.

TABLE S2 SNPs missing rate and heterozygosity of single genotypes and various populations (the European–American groups include the hybrid ‘Isabel’ and the hybrid ‘Seibel 128’ identified among the putative *sylvestris*); SNPs ancestry values for genetic groups inferred by STRUCTURE analysis on 13080 SNPs (SNPs-B matrix) including 37 *sylvestris*, 70 *sativa*, 3 non-*vinifera*, and 4 non-*Vitis* reference genotypes.

TABLE S3 SNPs matrix of 114 grapevine genotypes: 70 *Vitis vinifera* subsp. *sativa*, 37 *Vitis vinifera* subsp. *sylvestris*, 4 *Vitis* non-*vinifera*, 3 non-*Vitis.*
